# Age and gender dependent development of Theory of Mind in 6- to 8-years old children

**DOI:** 10.3389/fnhum.2013.00281

**Published:** 2013-06-17

**Authors:** Cecilia I. Calero, Alejo Salles, Mariano Semelman, Mariano Sigman

**Affiliations:** ^1^Laboratorio de Neurociencia Integrativa, Departamento de Física, Facultad de Ciencias Exactas y Naturales, Universidad de Buenos AiresBuenos Aires, Argentina; ^2^IFIBA, CONICETBuenos Aires, Argentina; ^3^Universidad Torcuato Di TellaBuenos Aires, Argentina

**Keywords:** Theory of Mind, scaling, mental states, development, gender differences

## Abstract

The ability to attribute different mental states to distinct individuals, or Theory of Mind (ToM), is widely believed to be developed mostly during preschool years. How different factors such as gender, number of siblings, or coarse personality traits affect this development is not entirely agreed upon. Here, we introduce a computerized version of the scaled ToM suite of tasks introduced by Wellman and Liu (2004), which allows us to meaningfully test ToM development on children 6 to 8-years old. We find that kids this age are still not entirely proficient in all ToM tasks, and continue to show a progression of performance with age. By testing this new age range, too, we are able to observe a significant advantage of girls over boys in ToM performance. Other factors such as number of siblings, birth order, and coarse personality traits show no significant relation with the ToM task results. Finally, we introduce a novel way to quantify the scaling property of the suite involving a sequence of set inclusions on one hand and a comparison between specially tailored sets of logistic models on the other. These measures confirm the validity of the scale in the 6- to 8-years old range.

## Introduction

Theory of Mind (ToM) is an important cognitive skill that refers broadly to our capacity to understand others' mental states including beliefs, desires, and knowledge, and the ability to comprehend that these may differ from our own (Premack and Woodruff, 1978). A paradigmatic example of a task requiring a well-developed ToM is that of false belief, which involves understanding that an agent might have a wrong representation of external reality, and act according to that representation (Baron-Cohen et al., 1985). ToM is deeply integrated with other cognitive domains and abilities. Among other factors, ToM development has been related to the success on executive function tests, and various assessments of language and social experience (Dunn et al., 1991; Dunn, 1995; Astington and Jenkins, 1999; Carlson and Moses, 2001; Apperly, 2012).

Over the past two decades, developmental changes in children's understanding of others' minds have been the focus of intense research (Wellman and Woolley, 1990; Dunn et al., 1991; Gopnik and Slaughter, 1991; Flavell, 1999; Astington and Jenkins, 1999; Meltzoff, 1999; Wellman et al., 2001). Most of the work performed to date was done in preschool children (3 to 5-years old) and coarsely agrees in that the basic aspects of ToM are mostly developed within this age range [Bartsch and Wellman, 1995; see Flavell (1999), for a review]. However, some studies argue that ToM continues to develop and change throughout life (Bosacki and Astington, 1999; Apperly, 2012; Devine and Hughes, 2012; Moran, 2013). Rai and Mitchell's (2004) study has shown that there is still considerable instability in understanding false beliefs in 5-years old, especially when the false belief scenario is framed in relation to a person's conscious choice or decision, rather than a physical object. Furthermore, Dumontheil et al.' (2010) results suggest that ToM improves between late adolescence and adulthood and even if ToM tasks are passed by age 4; their data indicate that the interaction between understanding others' mind and executive functions continues to develop in late adolescence (Dumontheil et al., 2010). Bosacki and Astington (1999) used ambiguous social vignettes followed by questions to assess the understanding of particular aspects of other's mental states in a study with preadolescent children. Their study was conceived from a ToM perspective in order to quantify preadolescents' mentalizing abilities and their results partially support that, individual differences in preadolescents' ability to understand the thoughts and emotions of others would be related to their social competence (Bosacki and Astington, 1999).

Most studies on ToM have not addressed issues of gender, family environment, and measures of temperament. A slight advantage of preschool girls on emotion understanding and false belief task performance has been observed before (Banerjee, 1997; Charman and Clements, 2002; Walker, 2005), nevertheless, most previous studies have found no significant gender differences on ToM development (Hughes and Dunn, 1998; Charman and Clements, 2002; Walker, 2005; Mathieson and Banerjee, 2011; Devine and Hughes, 2012). On the other hand, in studies carried out in preadolescence, girls performed significantly higher on the social understanding task (ToM) than boys independent of vocabulary ability (Bosacki and Astington, 1999). These results support Hatcher et al.' (1990) findings, in which girls scored higher than boys on social understanding tasks across grades 4 through 12. Further, the recent study in adolescents by Ibanez et al. (2013) presents a model that shows the direct effect of empathy, sex, and fluid intelligence on ToM. Only recent studies have found some relations between scores on false belief tasks and preschool children's family environments (Perner et al., 1994; Farhadian et al., 2010). However, there is no consensus on whether the amount of siblings or the birth order influences the development of ToM (Azmitia and Hesser, 1993; Lewis et al., 1996; Ruffman et al., 1998; Cutting and Dunn, 1999; Farhadian et al., 2010).

A complementary aim of most developmental studies of ToM consists in understanding the sequential unfolding of abilities underlying a full ToM. To date there is consensus in the notion that a child will correctly judge a person's desires before she can correctly judge her beliefs, and that she will be able to grasp that an agent might have a belief different from her own first if she doesn't know the true state of affairs, and only later if she does know what reality really is like (false belief). Cutting and Dunn (1999), Wellman and Liu (2004), Wellman et al. (2001) and, more recently, Wellman et al. (2011) have investigated this progression of abilities. In particular, Wellman and Liu (2004) proposed a suite of ToM tasks, based upon a meta-analysis of the literature of ToM developmental studies, and tested it in children from 3 to 5-years of age. Their results suggest that the abilities underlying ToM are attained progressively, and can thus be tested individually by an ordered suite such that a child capable of correctly performing a certain task in the suite should also be able to correctly perform all preceding tasks. Wellman and Liu's scaled suite of tasks has been subsequently employed to pinpoint cultural differences in the development of ToM (Wellman et al., 2006, 2011; Shahaeian et al., 2011).

In this work, we implement and test a computer version of Wellman and Liu's (2004) ToM suite in children in the 6 to 8-years old range. The aim of this work is threefold: first, we test whether it is possible to use the suite to test ToM development in older kids and check if the scaling seen in preschoolers is still valid in our version of the test. The age range chosen in this work is sometimes overlooked in the literature, even if it has been shown that children do not understand metaphor or irony before the age of six to seven (Ackerman, 1981)—-two behaviors that entail the capacity to go beyond the literal meaning of a statement—- and that they cannot reliably distinguish jokes from lies before age 6 to 7-years (Sullivan et al., 1995). In accordance, our first hypothesis is that ToM progression of Wellman and Liu's suite will still be present in children 6 to 8-years old.

Second, given that gender differences might be expected in ToM, and in line with the gender intensification hypothesis (Hill and Lynch, 1983), which establishes that gender differences increase in time because of growing pressure to conform to traditional gender-role stereotypes, we hypothesized that in a slightly older group of children—-in relation to the usually age range explore in the literature (3 to 5-years old)—- gender may have an appreciable effect on ToM performance.

The third and final aim of this work is to develop a novel analysis to quantify the validity of the scaling in the suite. The method we use has two parts: one uses set inclusions to quantify the extent to which the data differ from a perfect scaling, while the other involves the comparison between specially tailored sets of logistic models. The difference in prediction power among these sets of models gives another measure of the scaling quality.

## Materials and methods

### Participants

Seventy-six first (36) and second (40) graders [mean age: 7-years and 3 months (86.5 months); range from 6-years and 1 month to 8-years and 7 months] participated in the study. There were 42 boys and 34 girls in the sample, all of a high socioeconomic status and attending a well reputed private bilingual school in Buenos Aires. The school in which the study was performed approved the research and all children's parents or legal guardians gave signed voluntary consent. The consent form, presented to the caregivers supplemented with a note which explained the procedure, was previously authorized by the *Centro de Educación Médica e Investigaciones Clínicas “Norberto Quirno*” (CEMIC)'s Ethical Committee.

### ToM suite

**Figure 1:**
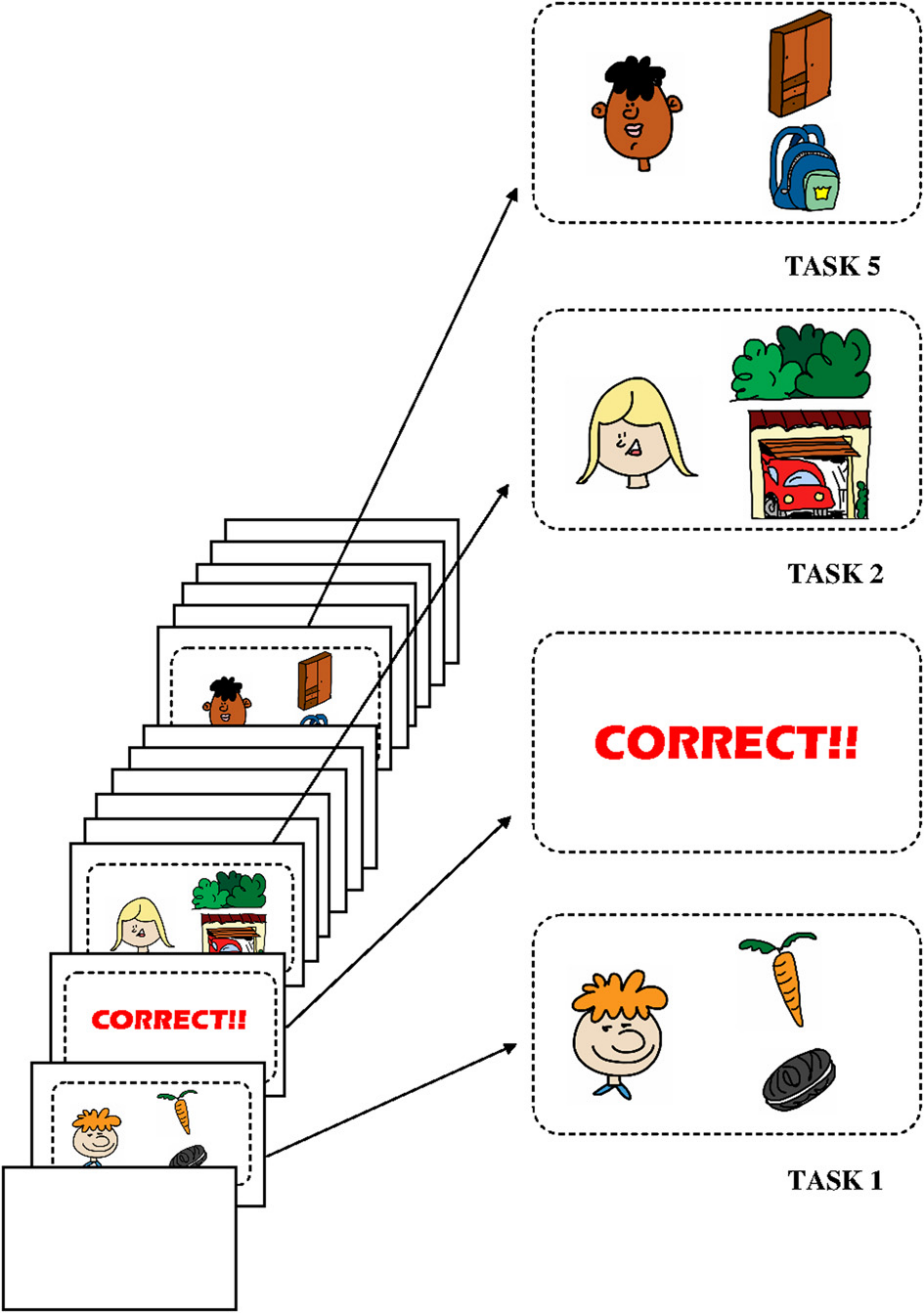
**Description of the test**. The test was delivered to each child in a novel computer format. The experimenter first said to each child: “There are six levels; you will have to answer correctly in each one of them to win the game. Let's go!” Then, for each task, the experimenter explained the task to the child, who could choose any of the options given. There were no wrong answers, such that, independently of the answer, a “CORRECT” screen appeared next. Three of the tasks are shown in the figure.

Wellman and Liu's ToM suite of tasks is thoroughly described in the appendix of the original paper (Wellman and Liu, 2004). Briefly, the tasks involved in our version are: (1, DD) Diverse Desires: the child judges that two persons (her vs. someone else) have different desires about the same objects; (2, DB) Diverse Beliefs: the child judges that two persons (her vs. someone else) have different beliefs about the same object, while she does not know which belief is the right one; (3, KA) Knowledge Access: the child sees what is in a box and judges the knowledge of another person who does not see what is in it; (4, FB) Contents False Belief: the child judges another person's false belief about what is in a distinctive container while she (the child) knows what actually is inside the container; (5, EFB) Explicit False Belief: the child judges how someone will search, given that person's mistaken belief, and (6, BE) Belief vs. Emotion: the child judges how a person will feel given a belief that is mistaken. All tasks involve a control question which is used to make sure that the child understood the task, and a target question, which evaluates their performance. Although all six tasks were used for studying the influence of diverse factors in ToM development, we note that only the first four (DD, DB, KA, and FB) are involved in the progressive suite, and thus all scaling tests were performed only on these. Finally, we note that Wellman and Liu's original version of the suite included also a hidden emotion task. As this task involves two target questions instead of one target and one control, we chose to leave it out in order to facilitate direct comparison (the random choice performance baseline for this task is 33% instead of the rest of the tasks' two choice 50%). The implementation of the suite is depicted in Figure 1.

In Wellman and Liu's version of the suite, some tasks were presented using toy figurines while others involved drawings. This makes it difficult both to preserve the parallels among the different tasks and to compare the results with those involving other methods found in the literature. Further, following our aim of carrying the scaled ToM suite to an older age range, we formulated it as an engaging computer game, which not only unifies the presentation format across all tasks, but also reduces the experimenter's involvement.

### Procedure

Children were tested in a quiet room in the school by one of two adult experimenters. The six tasks were presented in the increasing difficulty order proposed by Wellman and Liu (2004). All the children that participated in the study correctly answered the control question in each task.

Teachers completed the short form of the Child Behavioral Questionnaire (CBQ) for all their students. This allowed us to measure child's temperament along three broad dimensions: (1) Extraversion/Surgency, (2) Negative Affectivity, and (3) Effortful Control (Rothbart et al., 2001; Putnam and Rothbart, 2006). They also filled a second form that included the family background data (birth order and number of siblings), age, and gender of each child.

### Data analysis

In order to summarize the children's performance in the ToM suite we compute for each child her *z*-score, defined as the amount of correct target answers. Alternative measures were also tested, in which the contribution of each task to the total score was weighed either progressively with the task number or with a factor equal to one minus the observed mean performance for that task, and then all contributions summed together in a final score. The results obtained are insensitive to the scoring scheme chosen; we hence stick to the *z*-score above.

Throughout the analysis, we used non-parametric permutation tests in order to assess the significance of results. In each case, we randomly shuffled the assignments between predictor and dependent variables, to produce a surrogate version of the data. The relevant quantity (for instance correlation) was then evaluated for this surrogate data. By iterating this procedure many times (typically a thousand), we obtained the significance level of the result.

Apart from studying the correlations in the data, we built a logistic model to assess kids' responses for all tasks. With this model, we can study the effect of the different factors in the whole set of responses, without limiting the analysis to a particular definition of score. Nevertheless, a certain amount of independence among the factors entering the model is required in order to correctly interpret the results, and hence we restrict the model to age, gender, and number of siblings and birth order (considering these last two as a single factor in order to account for their interdependence). CBQ scores, on the other hand, were left out, since they correlate mildly with gender (ex. 0.4 linear correlation between surgency and gender).

Apart from gender, age, number of siblings, and birth order as predictors, the model includes dummy variables for discerning among the six tasks in the suite. Since each kid responds to all six tasks, we also need to index the subjects. As it turns out, age, sex, sibling amount, and sibling order taken together are almost enough to identify all subjects. There are however, five cases in which these repeat, so we resolve them by adding an extra indicator variable (i.e., order in which they took the experiment).

To measure the importance of each factor in predicting the results, we compare the full model with that with the factor in question removed. The difference between the log likelihoods of both models follows a χ^2^ distribution [apart from a factor of 2, see Stevenson (2004)], and we can hence evaluate the corresponding *p*-value. The *p*-values thus obtained are in agreement with those computed with the full model under the assumption of normally distributed errors.

## Results

### ToM performance in 6 to 8-years old children

**Figure 2:**
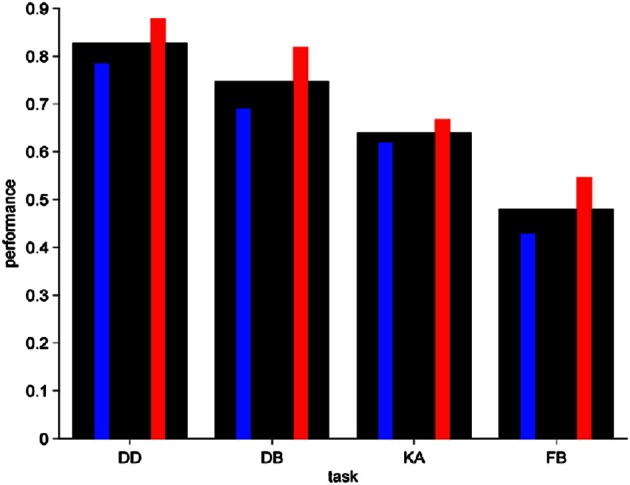
**Kids' average performance**. Fraction correct, mean, thick black bars; boys, left thin blue bars; girls, right thin red bars.

Despite the fact that some studies indicate that the development of a full ToM continues all throughout life (Devine and Hughes, 2012; Moran, 2013), almost all research has focused in 3 to 5-years old children. We took a step further to contribute to elucidate these notions and we examined age, gender, and family background influence on ToM development in the age range of 6 to 8 years old. Figure 2 shows the average performance for the four tasks involved in the scaling for all children (thick black bars). The average performance for our first and second graders is in the same range as that of the preschoolers studied by Wellman and Liu, suggesting that the smaller intervention of the experimenter enabled by the computer platform allows for the testing of older kids without saturating the suite. Average performance for tasks EFB and BE was 0.64 and 0.6, respectively, also similar to that of Wellman and Liu's preschoolers. These tasks were not included in the graph to emphasize the progression effect in the first four.

In accordance with our first hypothesis that ToM progression of Wellman and Liu's suite will still be present in 6 to 8-years old, we observed an increase in ToM proficiency with age, with *z*-scores significantly correlated with age (*r* = 0.334, *p* = 0.003 permutation test), This result stresses even further the fact that the test is effective in the new age range, and shows that the general performance drop is not merely due to a statistical fluctuation.

Average performance divided by gender is also shown in Figure 2 (boys in left blue thin bars and girls in right red thin bars). For task EFB we have average performances of 0.7 for girls and 0.6 for boys, while for the BE task we obtain 0.76 for girls and 0.48 for boys. We observe a clear effect of gender in ToM performance, with girls performing significantly better than boys in all tasks (*p* = 0.037 permutation test, grouped tasks). This gender effect is task-independent, as we show using the logistic model described in the following section.

We also studied the effect of family background and child temperament, factors whose potential influence in ToM performance has been previously discussed in the literature (Dunn et al., 1991; Perner et al., 1994; Dunn, 1995; Farhadian et al., 2010). Contrary to age and gender findings and in agreement with previous data (Lewis et al., 1996; Cutting and Dunn, 1999) we find no effect of sibling amount (*r* = 0.034, *p* = 0.766, permutation test) or birth order (*r* = −0.043, *p* = 0.729, permutation test) in ToM performance.

Similarly, coarse personality traits as evaluated by the CBQ do not correlate significantly with the *z*-score (Surgency: *r* = −0.169, *p* = 0.149; Negative Affect: *r* = 0.197, *p* = 0.093; Effortful Control: *r* = 0.118, *p* = 0.315; permutation tests).

The logistic model analysis fully supports the results discussed above. By taking one factor at a time out of the model, we can see how relevant each factor is in explaining the kids' responses (see Materials and Methods). The computed *p*-values are: age, *p* = 0.002; sex, *p* = 0.015; siblings (amount and order), *p* = 0.978. By testing an expanded model including a joint sex-task factor, we see that these two variables do not interact (*p* = 0.47). Hence, in accordance with our second hypothesis we observed a gender effect in ToM which was not observed for the same task in preschoolers. The other demographic or individual variables had no effect in performance.

### ToM suite progression

We now turn to quantify the extent to which the ToM suite embodies a progressive test, that is, one in which in order to correctly resolve a given step, one must have the abilities required to solve all previous steps. As we mentioned before, we only consider tasks 1–4 (DD, DB, KA, and FB) for this part of the analysis, since it is only these that are involved in the hierarchy.

The progression in difficulty of ToM performance is evident from Figure 2, which shows a diminishing performance with increasing task number. An average decrease in performance, however, does not necessarily imply a sequential process. It is still possible that a group of children is capable of successfully performing Task 1 (*G*_1_) and a smaller group is capable of successfully performing Task 2 (*G*_2_) but that these groups have no intrinsic relation other than their difference in size, i.e., |*G*_2_| < |*G*_1_|, where |·| denotes set cardinality. The condition of strict sequential dependence in performance implies that all kids in *G*_2_ are also in *G*_1_(*G*_2_ ⊂ *G*_1_), in other words, that children succeeding in Task 2 also succeed in Task 1. This logic extends for all tasks from 1 to 4, such that *G*_j_ ⊂ *G*_*i*_ for all *i* < *j* in 1 to 4.

**Figure 3:**
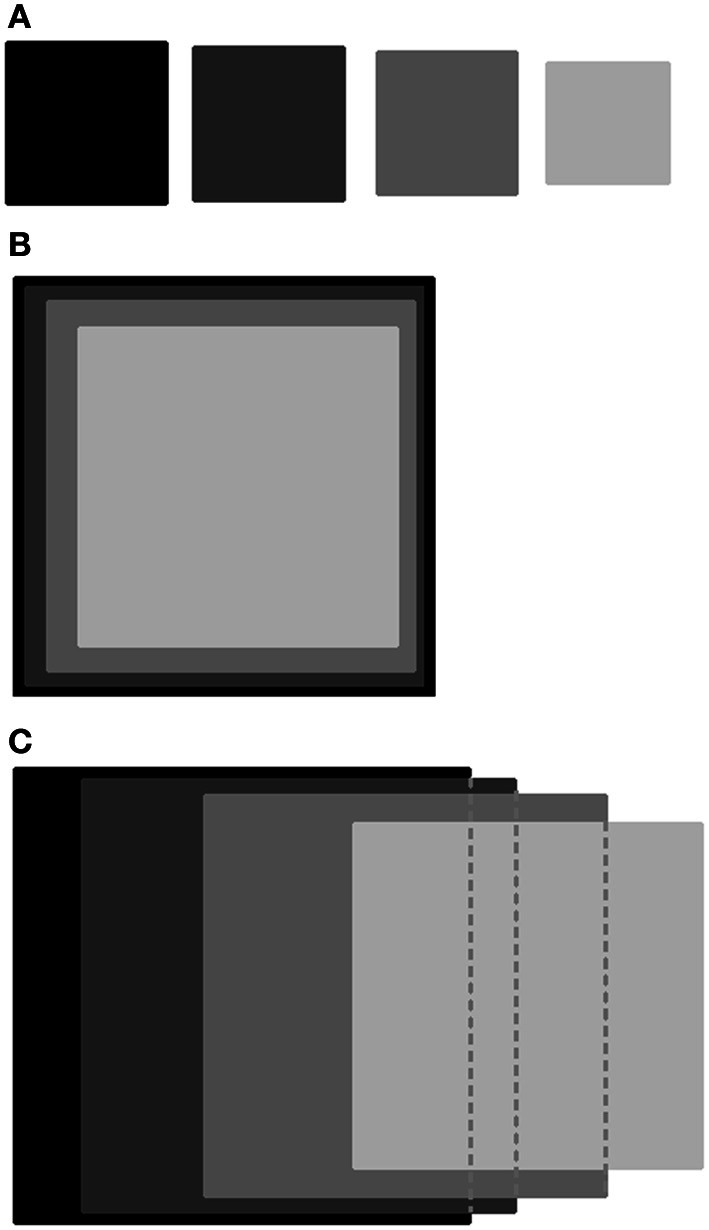
**Task progression as set inclusions**. Tasks DD, DB, KA, and FB are represented as squares in decreasing gray level. Square area represents amount of kids successfully completing each task, and area overlap among successive tasks quantifies the amount of kids that responded correctly for both tasks. **(A)** Illustrative lack of scaling. **(B)** Illustrative perfect scaling. **(C)** Actual experimental results.

Figure 3 depicts this sequence of inclusions for two extreme cases, **(A)** one in which there is no progression at all, and **(B)** one in which the hierarchy is perfect, along with our actual result **(C)**. In this figure, each task is represented by a square whose area encodes number of kids who successfully passed the task and area overlap indicates kids passing both corresponding tasks. For clarity, we only represent overlaps of successive tasks. Out of 76 kids, 62 passed DD, 56 passed DB, 48 passed KA, and 36 passed FB. Of these, 6 passed DB but not DD (11% of those that passed DB), 11 passed KA but not DB (23% of those that passed KA), and 10 passed FB but not KA (28% of those that passed KA). In a perfect hierarchy, there would be no such cases. These cases, however, constitute an expected fluctuation. In order to test this statistically, we take as a figure of merit the sum of cases in which a kid passed a task without passing the previous one, which is 27 in our case (lower values of this number represent better scalings). We then perform a bootstrap procedure in which we shuffle both kids and tasks, and obtain a surrogate value for this figure of merit. We find a better (lower) value in less than 1% of the cases, with an actual *p*-value of 0.059.

We refine this analysis by examining three sets of logistic models for the kids' responses, taken one task of the ToM suite at a time. The first is a set of Pointwise models, one for each task, which include as predictors age, gender and sibling amount and order. The second is a set of Markov models, again one for each task, which comprise besides the factors in the Pointwise models the response for the previous task in the scale. For the first task, these two models are identical. Finally, the third set is one of Cumulative models, also one for each task, incorporating the same predictors as the Pointwise models plus the whole history of previous responses for each kid up to the current task. We point out that for the second task of the scale the Markov and Cumulative models coincide.

We compare pairwise the model sets described before, getting a *p*-value for the extra variables in a model to be explanatory. The summarized results are presented in Table 1. We find that, comparing the Pointwise and Markov models, the response to the first task is a good predictor of the response to the second. This is consistent with the scaling property: a child that did not pass the first task is very likely to also not pass the second, hence making the Markov model more powerful than the Pointwise. We note however, that even in a perfect scaling, we would have some amount of unpredictability, since we do not know at which point the kids will start failing the tasks, so that even having the previous task information will not help in predicting the outcome of the next. This is indeed what we see when comparing the Markov and Pointwise models for the subsequent tasks, where more kids begin to fail.

**Table 1:** **Model sets comparison**.

**Task**	***p* Markov vs. Cumulative**	***p* Pointwise vs. Markov**
2 (DB)	–	0.006
3 (KA)	0.768	0.839
4 (FB)	0.724	0.322

We can address this issue by further comparing the Markov and the Cumulative model sets (different only for task 3 and up). This comparison shows that the extent to which one can predict the outcome in a certain task by knowing the result for the previous task is not improved by knowing further previous results, as should be the case in a perfect hierarchy. In other words, knowing the result for the first task does not add information to knowing the result for the second task, if we are to predict the response to the third one, and similarly for task 4. This further stresses the scaling property of the suite.

## Discussion

While many studies argue that by the age of four most normally developing children have already acquired an understanding of the mind; others instead have shown that ToM continues to mature at older ages Bosacki and Astington, 1999; Dumontheil et al., 2010; Devine and Hughes, 2012; Moran, 2013. In the present study we implement the ToM suite of tasks by means of a game in a computer platform, thus diminishing the experimenter's involvement. Corroborating Wellman and Liu (2004) previous results with preschool kids, we also found that the progressive and sequential effect of the suite could remain a major factor in older kids, revealing a hierarchy of nested processes of ToM in the 6 to 8-years range. However, because this ToM suite of tasks was never tested with the original age range and procedures in Argentina more studies need to be done.

We successfully apply the suite in older kids and to reach this conclusion we introduced a novel method to quantify the scaling property of the suite. This new proposal involves on one hand an intuitive quantification through set inclusions, and, on the other, a thorough comparison of a variety of logistic models including a varying amount of previous results as predictors for the outcome of a certain task. Both methods provide strong support for the scaling, and validate its use in the new age range tested.

Most studies on ToM have not addressed issues of gender. By testing older kids, we could examine the hypothesis that gender differences in ToM proficiency may develop late. This would be in accordance to the gender intensification hypothesis (Hill and Lynch, 1983), which predicts that gender differences increase in time because of increased pressure to conform to traditional gender-role stereotypes. Here, we conclusively showed a strong effect indicating that girls perform significantly better than boys for all ToM tasks in the age range tested.

Although some studies have proposed facilitative effects of (older) siblings that may operate via shared experiences of pretend play and deception, and talk about feelings and internal mental states (Perner et al., 1994; Ruffman et al., 1998; Peterson and McAlister, 2006), other work has found no relation between ToM performance an number of siblings or birth order (Cutting and Dunn, 1999; Cole and Mitchell, 2000; Hughes and Ensor, 2005). In our study, the family background included in the analysis did not correlate with ToM performance, contributing to the idea that birth order and number of siblings would not be related to the development of ToM. However, we need to take into consideration that our results come from children in a new age range (6 to 8-years old), while previous evidence related to this particular topic was concentrated on preschoolers.

Similarly, there is some evidence supporting a relation between understanding of false belief and emotion and peer-related social competence (Dunn et al., 1991; Dunn, 1995), although the individual influence of child temperament on ToM has not yet been studied. The relation between ToM performance and emotion and temperament is thus surprisingly unclear, despite the importance of both domains to social interactions (Cutting and Dunn, 1999). Only recent studies have found some relations between a direct relation of gender and ToM mediated by empathy (Ibanez et al., 2013). Here, we evaluated the relation between three personality traits as quantified by the CBQ and ToM performance. None of these traits appears to have an impact in the understanding of other's minds. We should point out, however, that given the lack of memory or general intelligence measure, the use of non-standardized measures and the ethnical homogeneity of our sample, among other factors, interpretations of the present findings are to be made with caution.

Finally, we introduced a novel way in which to quantify the scaling property of the suite. This new proposal involves on one hand an intuitive quantification through set inclusions, and, on the other, a thorough comparison of a variety of logistic models including a varying amount of previous results as predictors for the outcome of a certain task. Both methods provide strong support for the scaling, and validate its use in the new age range tested.

### Conflict of interest statement

The authors declare that the research was conducted in the absence of any commercial or financial relationships that could be construed as a potential conflict of interest.
